# Joint Effects of Known Type 2 Diabetes Susceptibility Loci in Genome-Wide Association Study of Singapore Chinese: The Singapore Chinese Health Study

**DOI:** 10.1371/journal.pone.0087762

**Published:** 2014-02-10

**Authors:** Zhanghua Chen, Mark A. Pereira, Mark Seielstad, Woon-Puay Koh, E. Shyong Tai, Yik-Ying Teo, Jianjun Liu, Chris Hsu, Renwei Wang, Andrew O. Odegaard, Bharat Thyagarajan, Revati Koratkar, Jian-Min Yuan, Myron D. Gross, Daniel O. Stram

**Affiliations:** 1 Department of Preventive Medicine, University of Southern California, Los Angeles, California, United States of America; 2 Division of Epidemiology and Community Health, University of Minnesota, Minneapolis, Minnesota, United States of America; 3 Department of Laboratory Medicine, Department of Epidemiology and Biostatistics, and California Institute for Quantitative Biosciences (QB3), University of California San Francisco, San Francisco, California, United States of America; 4 Duke-National University of Singapore Graduate Medical School, Singapore; 5 Saw Swee Hock School of Public Health, National University of Singapore, Singapore; 6 Human Genetics, Genome Institute of Singapore, A*STAR, Singapore; 7 Department of Epidemiology, University of Pittsburgh Cancer Institute, Pittsburgh, Pennsylvania, United States of America; Central China Normal University, China

## Abstract

**Background:**

Genome-wide association studies (GWAS) have identified genetic factors in type 2 diabetes (T2D), mostly among individuals of European ancestry. We tested whether previously identified T2D-associated single nucleotide polymorphisms (SNPs) replicate and whether SNPs in regions near known T2D SNPs were associated with T2D within the Singapore Chinese Health Study.

**Methods:**

2338 cases and 2339 T2D controls from the Singapore Chinese Health Study were genotyped for 507,509 SNPs. Imputation extended the genotyped SNPs to 7,514,461 with high estimated certainty (r^2^>0.8). Replication of known index SNP associations in T2D was attempted. Risk scores were computed as the sum of index risk alleles. SNPs in regions ±100 kb around each index were tested for associations with T2D in conditional fine-mapping analysis.

**Results:**

Of 69 index SNPs, 20 were genotyped directly and genotypes at 35 others were well imputed. Among the 55 SNPs with data, disease associations were replicated (at p<0.05) for 15 SNPs, while 32 more were directionally consistent with previous reports. Risk score was a significant predictor with a 2.03 fold higher risk CI (1.69–2.44) of T2D comparing the highest to lowest quintile of risk allele burden (p = 5.72×10^−14^). Two improved SNPs around index rs10923931 and 5 new candidate SNPs around indices rs10965250 and rs1111875 passed simple Bonferroni corrections for significance in conditional analysis. Nonetheless, only a small fraction (2.3% on the disease liability scale) of T2D burden in Singapore is explained by these SNPs.

**Conclusions:**

While diabetes risk in Singapore Chinese involves genetic variants, most disease risk remains unexplained. Further genetic work is ongoing in the Singapore Chinese population to identify unique common variants not already seen in earlier studies. However rapid increases in T2D risk have occurred in recent decades in this population, indicating that dynamic environmental influences and possibly gene by environment interactions complicate the genetic architecture of this disease.

## Introduction

T2D remains a very serious health threat in developed countries and is becoming a major health threat in many under-developed countries, particularly those with rapidly growing economies [1]–[3]. Globally, T2D affected over 360 million people in 2011 [4] and this number is projected to increase rapidly in upcoming years. This rise in risk is paralleled by a rapidly increasing incidence of obesity in many populations, a major risk factor for diabetes. In addition, incidence may be propelled by an elevated genetic susceptibility in some populations. Other risk factors include dietary patterns [5], [6], sedentary lifestyle [7], [8], psychosocial stress [9]–[11], short sleep hours [12], and smoking [13]–[16].

Interestingly, the prevalence of T2D is much higher (approximately 2-fold) in several Southeast or East Asian populations than in populations of European-descent, even though most Asians have a much lower average body mass index (BMI) and rates of obesity [17]–[20]. The prevalence of T2D continues growing rapidly in many Southeast Asian countries, including Singapore [21]. Compared to populations of European ancestry, East Asians, including Chinese and Japanese have been characterized as having a higher proportion of abdominal and visceral fat deposits in the presence of a BMI≤25 kg/m^2^ [22], [23], which is considered a healthy BMI in populations of European descent. Also, diabetes incidence in young to middle-aged people is disproportionately higher in Southeast Asia than in the West [21]. This apparent difference in susceptibility is recognized by the International Federation of Diabetes, which has established lower BMI cutoffs for overweight and obesity than are used for populations of European-descent [24]. The apparently higher susceptibility persists in individuals migrating from Southeast Asia to other parts of the world and results in even higher levels of diabetes in these populations when living in Western cultures [25]–[28].

It is well-known that T2D is heritable in many populations [29]–[31] and has a familial recurrence risk ratio for first degree relatives of approximately two [32], [33]. In addition, numerous studies have associated specific genetic variants with the risk of T2D. Several notable associations were identified by linkage analysis and candidate gene studies, and include *PPARγ* [34], *KCNJ11* [35], *WFS1* [36] and *TCF7L2* [37]. The advent of large-scale genetic studies searching the entire genome for common SNPs (frequency>5%) associated with diabetes has significantly increased the number of SNPs associated with diabetes. Since 2007, genome-wide association studies (GWAS) have reported at least 57 additional thoroughly replicated genetic susceptibility loci harboring common variants for T2D [38]–[57]. Most of these were novel disease loci and contributed to a better understanding of diabetes heritability. However, the effect sizes of these loci were small and only a small proportion of the heritability of T2D was explained [58]. Moreover, most of the SNP associations discovered by GWAS were identified in European populations. However, Asian-specific SNPs have been identified and several loci were first identified by GWAS in Asians including *KCNQ1* [40], [59], *UBE2E2* and *C2CD4A-C2CD4B* [48].

We investigated the reproducibility of single SNP associations in a study of T2D among Singapore Chinese using both genotyped and imputed alleles. Beyond investigating associations between single variants and disease risk, it is important to consider the combined effects of various loci on disease risk. In this report, we used the National Human Genome Research Institute (NHGRI) GWAS Catalog [60] to identify 59 single-nucleotide polymorphisms (SNPs) in 46 gene regions that have been associated with T2D. In addition we interrogated regions near GWAS alleles to search for additional or refined associations.

## Research Design and Methods

### Ethics Statement

This study has been approved by the institutional review boards of the National University of Singapore, the University of Southern California, the University of Minnesota, and the University of Pittsburgh. Informed written consent to participate in biomarker studies was obtained at time of specimen collection. The institutional review boards approved this consent procedure.

### Study Population

People of Chinese ancestry comprise the largest ethnic group in Singapore and constitute 74.1% of Singapore's resident population [61]. The design of the Singapore Chinese Health Study has been previously described [62]. Briefly the cohort is drawn from permanent residents or citizens of Singapore aged 45–75 at study entry, who reside in government-built housing estates (∼86% of Singapore residents live in such facilities). Migration out of Singapore, especially among housing estates residents is negligible (Department of Statistic, Singapore Ministry of Trade and Industry, 1997). The study subjects are restricted to the two major dialect groups of Chinese in Singapore: The Hokkiens, who originated from southern Fujian Province, and the Cantonese, who came from Guangdong Province (Both provinces are in south eastern China. The gender dialect breakdown of the cohort is as follows, 15,617 (24.7%) Hokkien men, 18,356 (29.0%) Hokkien women, 12,342 (19.5%) Cantonese men, and 16,942 (26.8%) Cantonese women.

Between April 1993 and December 1998, 63,257 individuals completed an in-person interview that included questions on usual diet, demographics, height and weight, use of tobacco, usual physical activity, menstrual and reproductive history (women only), medical history, and family history of cancer. A follow-up telephone interview took place between 1999 and 2004 for 52,325 cohort members (83% of recruited cohort). Beginning in April 1994, a random 3% sample of cohort participants were asked to provide blood or buccal cells, and spot urine samples. Eligibility for this biospecimen subcohort was extended to all surviving cohort participants starting in January 2000. By April 2005, all surviving cohort subjects had been contacted for biospecimen donation. Samples were obtained from 32,535 subjects, representing a consent rate of about 60%. The institutional review boards at the National University of Singapore, the University of Minnesota, and the University of Pittsburgh approved this study.

Utilizing resources of the Singapore Chinese Health Study, we conducted a genome-wide association study (GWAS) for the risk of developing diabetes that has a two staged design in which approximately 1/2 of all participants in the study are genotyped using a GWAS array with the remaining subjects genotyped as a replication study of the top SNPs found in stage 1. This approach follows the general principles of Satagopan et al [63] and Wang et al [64]. Herein we report results from the first stage of this study focusing on replication and fine-mapping of already-discovered genetic variants.

### Ascertainment of Type 2 Diabetes

For each study participant, the history of physician-diagnosed diabetes was asked at a baseline interview administered by a trained interviewer. Diabetes status was assessed again by the following question asked during the first and second follow-up telephone interviews: “Have you been told by a doctor that you have diabetes (high blood sugar)?” If yes: “Please also tell me the age at which you were first diagnosed”. The prevalent diabetes cases were those who reported a history of diabetes at the baseline interview whereas the incident diabetes were those reporting the initial diagnosis of diabetes that took place after the baseline interview in either the follow-up I or follow-up II interview (∼5.5 years between interviews). A validation study of the incident diabetes mellitus cases used two different methods and was reported in detail previously [65], [66]. Based on a hospital-based discharge summary database and a supplementary questionnaire regarding symptoms, diagnostic tests and hyperglycemic therapy during a telephone interview we observed a positive predictive value of 99% [66]. In other words, the self-reported history of diabetes was a highly reliable measure of diabetes status of the study population.

### Eligible Study Subjects

The cohort participants who did not report a history of diabetes at baseline interview and donated blood samples were eligible for the present study. We excluded subjects with prevalent diabetes at the baseline interview (n = 2,080) or did not provide blood samples (n = 36,245). The present study was based on the remaining 24,932 subjects. Among them, we identified 1,284 incident diabetes cases during the follow-up I interview in 2000–2005, and an additional 1,343 incident diabetes cases during the follow-up II interview in 2006–2011. For each incident diabetes case, one control subject was randomly selected among the subjects that provided blood samples but did not have a history of diabetes. Controls were matched to the index cases on gender, dialect group (Cantonese or Hokkien), age at baseline interview (±3 years), year of baseline interview (±2 years), and date of blood draw (±6 months). In addition, the selected controls were screened for the presence of undiagnosed T2D. The criterion for undiagnosed diabetes was the hemoglobin A1c (HbA1c)≥6.0%. All matched controls with HbA1c≥6.0% were ineligible for the study and a replacement control with the same matching criteria was randomly chosen among the remaining eligible subjects. Blood for HbA_1c_ analysis was collected in EDTA (ethylenediaminetetraacetic acid) tubes. Red-blood cells (RBCs) were isolated from whole blood and frozen until analysis was performed at University of Minnesota, a Clinical Laboratory Improvement Amendments (CLIA)-certified laboratory. HbA1c was measured with a dedicated HPLC instrument in our laboratory which serves as a reference laboratory for this assay. The instrument, a TOSOH HPLC, utilizes ion-exchange chromatography (Tosoh A1c 2.2 Plus HPLC, Tosoh Medics, Inc., Foster City, CA). This instrumentation is also referred to as the Tosoh G7/G8 HPLC Glycohemoglobin Analyzer (Tosoh Medics, Inc., San Francisco, California). A small red blood cell sample was automatically hemolyzed prior to injection onto the column. The labile fraction is separated on-line as a distinct peak and excluded from the calculation of % HbA1c. The hemoglobin fractions (A1a, A1b, F, Labile A1c, Stable A1c, A0 and Hb variants) are separated by a buffer gradient of increasing ionic strength. The Tosoh 2.2+ was calibrated daily using 2 calibrators (2-point calibration) standardized to a reference system and the percentage of HbA1c was calculated based on this system. Using the standards developed in the National Glycohemoglobin Standardization Program, this method was calibrated to the reference range of 4.3%–6.0% and had a laboratory coefficient of variation range 1.4%–1.9% [67].

### Genotype Analysis and Quality Control

Peripheral blood samples from 2615 incident diabetes cases and 2615 matched controls were selected for DNA extraction in stage 1. The DNA extraction was conducted at the Molecular Epidemiology and Biomarker Research Laboratory at the University of Minnesota (approximately 2/3rds of the samples) or the Genome Institute of Singapore (approximately 1/3rd of the samples) using the Qiagen method. DNA concentrations were measured by the PicoGreen and Nanodrop methods and prepared for genotype analysis.

Stage 1 genotyping was performed at the Genome Institute of Singapore according to the manufacturer's recommendations using an Affymetrix ASI (Asian) Axiom array. Genotype calling was performed by the Affymetrix Corporation. A standard series of QC steps were followed in order to identify SNPs in case and control samples for genetic association analyses. Starting with 510,584 SNPs provided for 4,918 callable study samples, we excluded samples with SNP call rates of less than 98 percent (n = 22) and SNPs (n = 3,075) with call rates less than 98 percent, leaving 507,509 SNPs.

We estimated relatedness between pairs of samples as the expected number of alleles shared identically by descent, *r*_ij_, using PLINK [68]. We dropped two pairs of unintended duplicate samples that were discovered to have *r*_ij_ close to one; we also dropped samples that appeared to be closely related (*r*_ij_>.2) to more than one other sample in the study and one of each remaining pair of samples with *r*_ij_>.2 (n = 180 total including the duplicates). We compared reported sex of each sample to sex as inferred on the basis of X chromosome heterozygosity, dropping 29 uncertain or conflicting samples. We computed principal components of the genotype matrix and dropped 9 individuals who were more than 5 standard deviations from the mean on any of the first 4 principal components. One additional sample was dropped because of missing covariate information. A total of 4,677 samples (2338 cases and 2339 controls) remained after QC analysis.

### Statistical Analysis

Characteristics of the cohort were compared between diabetes cases and controls. Two sample t-tests were used to compare the mean differences for variables with normal distributions. The Wilcoxon rank sum test was used to compare median differences for variables with skewed distributions. Pearson χ^2^ was used to test if the frequency distributions for categorical variables were different between diabetes cases and controls.

For genotype imputation, we first mapped genetic positions of our GWAS data to NCBI build37 using UCSC Genome Browser liftOver [69]. 14,032 (<3%) SNPs failed to be mapped to NCBI build37. The Segmented Haplotype Estimation and Imputation tool (SHAPEIT) [70] was then used to phase the remaining 493,477 SNPs. We applied 1000 Genomes Project Phase I data “version 3” [71] as the reference panel, which contained 1092 individuals of various ethnicities (246 Africans, 181 African Americans, 286 East Asians and 379 Europeans) with 36,648,992 SNPs. IMPUTE2 [72] was run to perform the imputation, which extended our total SNPs to be 36,617,842. After filtering out SNPs imputed to be monomorphic or with estimated r^2^<0.8, there were 7,514,461 imputed or genotyped SNPs for association analysis.

For this report, we selected 83 SNPs associated with T2D summarized by NHGRI GWAS Catalog [60] and significantly associated with diabetes risk at a well-recognized criteria for genome-wide significance (p≤5×10^−8^). Among these, one SNP was neither genotyped nor imputed in our data, 12 SNPs were poorly imputed with estimated certainty r^2^<0.8, and one genotyped SNP had rare minor allele frequency (MAF) less than 0.008. Additionally, 14 of the GWAS SNPs were found to be in LD with 11 other GWAS SNPs with estimated pairwise r^2^>0.75 using our genotyped and imputed data. After excluding these 28 SNPs, the logistic regression method was used to analyze the single SNP associations of the remaining 55 GWAS-implicated SNPs with diabetes case-control status after adjusting for age, sex, dialect, and first 10 principal components. The logistic regressions utilized the observed genotyped or expected imputed allele counts as the explanatory variable of interest.

The 55 SNPs from the GWAS catalog are called “index SNPs” in the fine-mapping analysis. Among these 55 SNPs, one SNP had no reported risk allele in the GWAS-catalog and the original papers. Thus, it was not included in the genetic risk score analysis described below. Power calculations were conducted using Quanto [73] for the 55 SNPs based on the risk allele frequencies in our 4,677 study subjects using a significance level of 0.05 and the odds ratio reported by the GWAS-catalog.

After single SNP association analysis, we constructed genetic risk scores based on genotyped only, imputed only, and both genotyped and imputed known diabetes SNPs combined by adding the observed or expected number of risk alleles for each study participant according to the risk allele reported in GWAS Catalog. The association between the genetic risk score and diabetes mellitus status was assessed using logistic regression adjusting for the same covariates as in the previous single SNP association analysis.

For fine-mapping analysis, regions 100 kb up and down stream of each index SNP were obtained from the combination of genotyped and imputed data. As before, logistic regression was used to test significant associations between the observed or expected allele counts (log additive model) for each SNP and disease status. Additionally, conditional analysis was performed for each SNP in a GWAS-indicated region by adjusting for the index SNP of that region in addition to the other covariates. Such conditional analysis attempts to refine SNP associations and search for stronger signals than index SNPs. Bonferroni adjustment was used to set the significance level for SNP association tests as 0.05/number of SNPs in each region. Based on fine-mapping results and following the approach of Chen et al [74], we attempted to define two types of SNPs: 1. “Improved SNPs”, i.e. SNPs in LD (in the original populations) with index signals (r^2^≥0.5) but with stronger results in the present GWAS than the index signals; 2. “New SNPs” i.e. SNPs with significant associations, but which were not in close LD with index signals (r^2^<0.5) that may reflect new associations in regions already known to be involved in disease risk. Next, fine-mapping results were used to improve the genetic score by substituting index SNPs with improved SNPs and adding new SNPs into the score. The risk allele and effect sizes of improved SNPs as well as new SNPs were defined based on our fine-mapping results.

Finally, Genome-wide Complex Trait Analysis (GCTA) was performed to estimate the proportion of disease variance (using a liability model) that is explained by GWAS reported diabetes SNPs as well as any newly identified SNPs from the fine-mapping analysis [75].

## Results

Characteristics of subjects in this study are presented in Table 1. The mean age and distributions of female gender, dialect group and smoking status or duration of smoking in cases were comparable with those in controls. Compared to controls, cases had a higher BMI (p<0.0001) and lower level of education (p = 0.004). More controls had weekly engagement of physical activities than cases (p = 0.043).

**Table 1:** Characteristics of subjects comparing T2D cases and controls.

	Cases (n = 2338)	Controls (n = 2339)	p
*Anthropometrics*			
Age [yrs]*	55.3 (7.2)	55.2 (7.1)	0.575
Females‡	1227 (52.5)	1247 (53.3)	0.568
BMI [kg/m^2^]*	24.6 (3.4)	22.7 (3.1)	<0.0001
Physical activity weekly (%)‡	32.7	35.5	0.043
Food energy intake [kcal/day]*	1619 (589)	1578 (547)	0.014
*Smoking History*			
non-smokers‡ §	1579 (69.3)	1643 (71.9)	
Former smokers‡ §	285 (12.5)	258 (11.3)	0.143
Current smokers‡ §	415 (18.2)	383 (16.8)	
Years of smoking (among current and former smokers) † §	25 (15, 35)	25 (15, 35)	0.808
*Education levels*			
No education‡	543 (23.2)	483 (20.7)	
Primary school education‡	1074 (45.9)	1033 (44.2)	0.004
Secondary school education or more‡	721 (30.8)	823 (35.2)	
*Dialect group*			
Cantonese‡	1166 (49.9)	1165 (49.8)	0.965
Hokkien‡	1172 (50.1)	1174 (50.2)	

* Variables are presented as mean (standard deviation). Two-sample independent t-test is used to test the mean differences between cases and controls.

† Variables are not normally distributed and are presented as median (25^th^, 75^th^ percentiles). Wilcoxon rank-sum test is used to compare the median differences between cases and controls.

‡ Categorical variables are presented as frequencies (%).χ^2^ test is used to test whether the distribution between cases and controls is different.

§ Variables have 59 cases and 55 controls missing.

**Figure 1:**
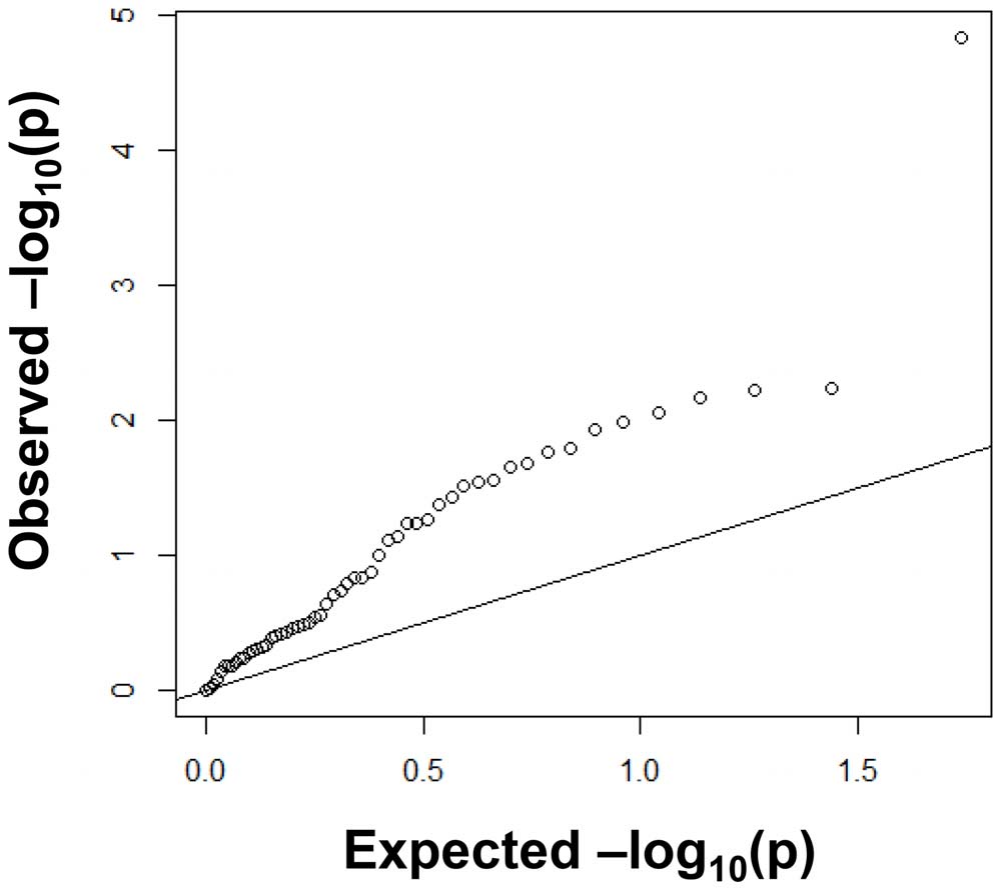
QQ-plot of 55 T2D SNPs reported by GWAS-catalog. Observed distribution of −log *P*-values were compared to the expected (null) distribution.

Among 55 potentially diabetes-related SNPs identified from the NHLBI GWAS Catalog, 20 SNPs were genotyped and 35 SNPs were imputed with r^2^>0.8. Here r^2^ was estimated as sample variance (over all individuals in the study) of the expected allele count (i.e. the imputed values) divided by the theoretical value, *2p*(*1-p*), of the variance of the count for a SNP in Hardy Weinberg equilibrium where *p* is the estimated frequency of the allele [76]. Based upon the risk allele frequency seen in our sample from the Singapore Chinese Health Study and on the reported odds ratio and risk allele from the GWAS catalog (for 54 SNPs with this information available) we had an average of 62.8 percent power to replicate true associations at a 5 percent significance level. Of the 54 SNPs with known risk alleles we found that 15 (27.8%) had significant associations (p<0.05) in the same direction as those reported with diabetes risk after adjusting for age, sex, dialect and 10 principal components (Table S1). Among the remaining 39 non-significant associations a total of 32 (82.1%) of the associations indicated that the same allele was associated with increased risk as listed in the GWAS catalog. Quantile-quantile (QQ) plots (Figure 1) of the p-values for association of the 55 SNPs showed considerable deviation from the distribution expected under the null hypothesis, further indicating that these 55 index SNPs included strong signals for diabetes risk in the Singapore Chinese population.

Non-replication of known or putative disease SNPs may be a result of differing LD patterns in Singapore Chinese relative to the original GWAS populations so that index SNPs might not be sufficiently correlated with the underlying biological causal variant in Singapore Chinese. In order to try to identify better genetic markers of risk in Singapore Chinese, we conducted fine-mapping analysis across all risk regions (±100 kb of index SNP), using genotyped SNPs on the Affymetrix array and imputed SNPs seen in the 1000 Genomes data (see Methods).

**Figure 2:**
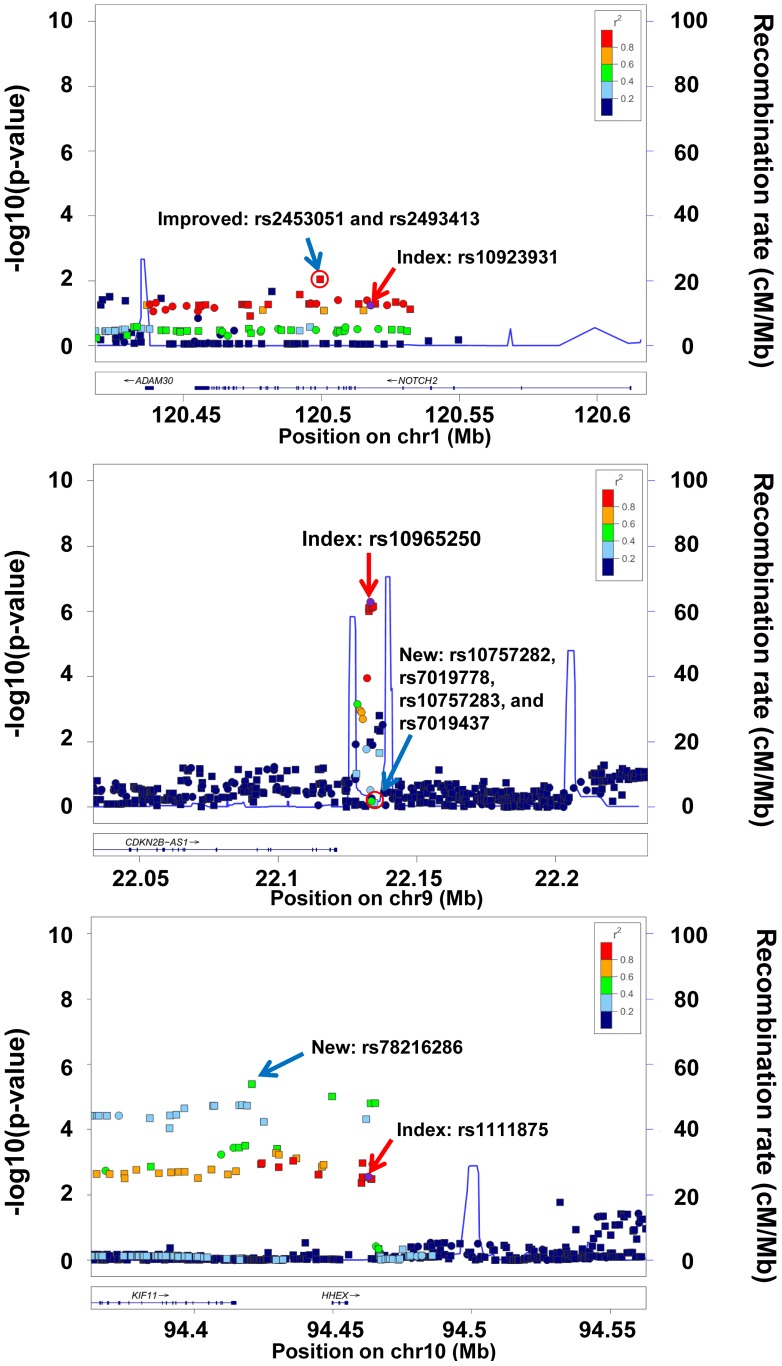
−log *P* -plots of the improved and novel T2D signals around index SNPs: rs10923931, rs10965250 and rs1111875 found by fine-mapping analysis. −Log *P*-value for risk-associated allele from the logistic regression model adjusted for age, sex, dialect and global ancestry (the first 10 principal components). Pairwise correlations (r^2^) in the 1000 Genomes Asian population are shown in relation to markers identified through fine-mapping in our sample. Squares denote genotyped SNPs; circles, imputed SNPs. Gray squares and circles denote that r^2^ cannot be estimated (not in 1000 Genomes). Red arrows and diamond denote the index SNP. Blue arrows denote the novel signal. The plots were generated using LocusZoom [86].

We searched for improved candidate SNPs from among those 1000 genome SNPs that were found to be in high LD (r^2^≥0.5) in the original GWAS population, as well as for novel SNPs not highly correlated with the index within the reported regions. After applying a Bonferroni correction for the number of SNPs tested in each region, we found two improved signals (both Bonferroni adjusted p-values = 0.033, Figure 2) for rs2453051 and rs2493413 having r^2^ = 1 (in Europeans based on 1000 genomes pilot data) with index SNP rs10923931. The two improved signals and the index SNP were located in the *NOTCH2* gene on chromosome 1. Additionally, we found five novel independent associations in 2 regions. Four correlated (pairwise r^2^>0.97) novel SNPs (rs10757282, rs7019778, rs10757283, and rs7019437) were found around index rs10965250 (Bonferroni adjusted p-value<0.044 for all, Figure 2). These SNPs were on chromosome 9 and near the *N2B-AS1* gene. SNP rs10757282 had the most significant association (Bonferroni adjusted p-value = 0.028). Another three significant associations (rs11187139, rs10882102 and rs78216286) were found around index rs1111875 (Bonferroni adjusted p-value<0.040 for all, Figure 2), however two of these SNPs rs11187139 and rs10882102 were on closer inspection found to be correlated with another nearby index SNP rs5015480 (r^2^>0.84), thus are not included in further analysis. The remaining SNP rs78216286 was on chromosome 10 near the *KIF11* gene. SNP rs78216286 is included in the following risk score analysis. These novel signals may indicate additional causal variants unidentified in the original GWAS.

The cumulative effect of all T2D risk variants was tested using unweighted counts of all diabetes risk SNPs. We did association analysis using a risk score comprised of four sets of risk alleles: 1) 19 genotyped SNPs; 2) 35 imputed SNPs; 3) 54 SNPs (genotyped and imputed); 4) original 54 SNPs with rs10923931 replaced by rs2453051, and including 2 new independent SNPs identified from fine-mapping analysis (rs10757282, and rs78216286) (Table 2). Using the 54 index SNPs from the GWAS catalog, the risk per allele was 1.049 (95% confidence interval (CI) 1.036–1.062; p = 2.93×10^−14^). Individuals in the highest quintile of the risk allele distribution were at 2.0-fold greater risk (p = 5.72×10^−14^) of T2D compared to individuals in the lowest quintile (Table 2). In single SNP analysis for the genotyped SNPs the mean odds ratio in the Singapore data was 1.100 while for the imputed SNPs the mean odds ratio was 1.058. In the risk score using genotyped SNPs the estimated OR per allele was 1.073 (1.049–1.097; p = 4.30×10^−10^). For the risk score with only imputed SNPs the odds ratio per allele was OR = 1.048 (95% CI: 1.031–1.065; p = 3.19×10^−8^). When the three new or improved SNPs were included in the risk score the association with T2D was slightly strengthened (per allele OR = 1.053; 95% CI 1.040–1.066; p = 6.68×10^−16^). Compared to individuals in the lowest quintile of this risk score, those in the highest quintile had a 2.1 times greater risk of the disease (p = 2.09×10^−16^). Interestingly we noted no evidence that the per allele odds ratios were different depending upon whether the index SNP was reported in GWAS of either a European or Asian population (mean OR in the Singapore sample was 1.063 for the 19 SNPs reported from GWAS in Asian populations versus 1.078 for the 35 SNPs reported from GWAS in European populations, Table S1).

**Table 2:** Summary risk scores in association with T2D.

		2,338 cases, 2,339 controls
Summary score of genotyped index markers (19 markers)		
Mean risk score of cases (range)/controls (range)		14.8 (7.0–25.0)/14.3 (6.0–25.0)
Per allele OR		1.073 (1.049–1.097)
P_trend_		4.301×10^−10^
Quintile OR (95% CI)	Q1	1.00 (ref)
	Q2	1.26 (1.03–1.53)
	Q3	1.40 (1.19–1.65)
	Q4	1.28 (1.04–1.56)
	Q5	1.68 (1.41–1.99)

Summary score of well imputed index markers (35 markers)		
Mean risk score of cases (range)/controls (range)		37.1 (24.3–48.8)/36.5 (21.5–47.8)
Per allele OR		1.048 (1.031–1.065)
P_trend_		3.193×10^−8^
Quintile OR (95% CI)	Q1	1.00 (ref)
	Q2	1.19 (0.99–1.43)
	Q3	1.36 (1.14–1.64)
	Q4	1.32 (1.10–1.59)
	Q5	1.66 (1.38–1.99)

Summary score of all imputed index markers (44 markers)		
Mean risk score of cases (range)/controls (range)		49.4 (36.3–61.7)/48.7 (33.9–60.9)
Per allele OR		1.048 (1.032–1.064)
P_trend_		1.172×10^−9^
Quintile OR (95% CI)	Q1	1.00 (ref)
	Q2	1.04 (0.87–1.25)
	Q3	1.27 (1.06–1.52)
	Q4	1.37 (1.14–1.64)
	Q5	1.57 (1.31–1.89)

Summary score of genotyped and well imputed index markers (54 markers)		
Mean risk score of cases (range)/controls (range)		51.9 (36.8–66.9)/50.8 (33.5–70.5)
Per allele OR		1.049 (1.036–1.062)
P_trend_		2.931×10^−14^
Quintile OR (95% CI)	Q1	1.00 (ref)
	Q2	1.39 (1.16–1.67)
	Q3	1.34 (1.12–1.61)
	Q4	1.57 (1.31–1.89)
	Q5	2.03 (1.69–2.44)

Summary score of all index markers (63 markers)		
Mean risk score of cases (range)/controls (range)		64.2 (47.3–80.4)/63.0 (45.9–80.1)
Per allele OR		1.050 (1.037–1.062)
P_trend_		1.147×10^−15^
Quintile OR (95% CI)	Q1	1.00 (ref)
	Q2	1.26 (1.05–1.52)
	Q3	1.23 (1.02–1.48)
	Q4	1.57 (1.31–1.89)
	Q5	2.03 (1.69–2.44)

Summary score of genotyped, well imputed index markers and 2 novel marker (56 markers)		
Mean risk score of cases (range)/controls (range)		53.3 (38.3–68.2)/52.2 (36.1–70.5)
Per allele OR		1.053 (1.040–1.066)
P_trend_		6.679×10^−16^
Quintile OR (95% CI)	Q1	1.00 (ref)
	Q2	1.27 (1.06–1.52)
	Q3	1.25 (1.04–1.50)
	Q4	1.60 (1.33–1.92)
	Q5	2.10 (1.74–2.52)

Summary score of all index markers and 2 novel marker (65 markers)		
Mean risk score of cases (range)/controls (range)		65.6 (49.9–81.4)/64.4 (48.9–80.1)
Per allele OR		1.053 (1.040–1.065)
P_trend_		2.684×10^−17^
Quintile OR (95% CI)	Q1	1.00 (ref)
	Q2	1.30 (1.09–1.57)
	Q3	1.36 (1.13–1.63)
	Q4	1.58 (1.31–1.90)
	Q5	2.18 (1.81–2.62)

Finally, we estimated the proportion of variance of diabetes risk (on the liability scale) explained by these SNPs using the GCTA program [77]. We assumed the prevalence of diabetes among the population to be 0.08 based on International Diabetes Federation report [4] and found that the 55 GWAS-reported diabetes SNPs explained 2.3% of disease liability variance after adjusting for age, sex, dialect and first 10 principal components (p = 0.007). After adding two novel SNPs from our fine-mapping analysis, the entire 57 SNPs were again estimated to explain 2.3% variance of the liability of diabetes in the sample (p = 0.007).

## Discussion

Replication and fine mapping of GWAS index disease associations in additional populations is useful for defining the relevancy of associations discovered in one population to other ethnic groups. In addition, studies of ethnically diverse groups contribute to the localization of associations and the discovery of new disease risk alleles in previously identified regions [74].

We were able to replicate disease associations (p<0.05) for 15 of 54 SNPs considered validated by prior studies. Of the 39 SNPs that were not replicated at p<0.05, the average power based on the GWAS-reported OR and Singapore Chinese risk allele frequency was 58.5 percent (Table S1, Figure S1) compared to 73.8 percent for the replicated SNPs. Our failure to replicate more known associations, despite reasonable power to do so, may be due to several reasons; it is possible that the odds ratios estimated for the reported risk alleles were biased upwards by a “winner's curse” phenomenon [78] thus causing an overestimation of statistical power for replication. Our risk score analysis using the sum of all 54 (both genotyped and well imputed) GWAS-significant risk alleles as a predictor of T2D risk in the Singapore Chinese Health Study population, while highly significant statistically (p<10^−13^), showed per-allele ORs that are smaller on average (1.05) than the mean (1.16) of the published ORs for these alleles or of the mean (1.07) of the single SNP ORs estimated in this study. This appears to be indicating either a sub-multiplicative effect of the SNPs in aggregate and/or reflecting a slight negative correlation (r = −0.25) between risk allele frequency and OR evident in Table S1. Additionally including nine poorly imputed SNPs into the risk score did not significantly influence previous results (per-allele ORs = 1.05, 95% CI: 1.04–1.06, p = 1.147×10^−16^, Table S1).

The attenuation of effect between the reported ORs and the ORs estimated here may also be due to differences in LD between the initial GWAS populations and the Singapore Chinese so that the correlation between index SNP and underlying causal variant is lower. In fine mapping analysis we found an improved signal for two SNPs (rs2453051 and rs2493413) that were in high LD with the index SNP rs10923931 in the original (European) reporting population but not in our study (r^2^ = .426). We also found five novel candidate SNPs (rs10757282, rs7019778, rs10757283, rs7019437, rs78216286) near two index SNPs, rs10965250 and rs1111875, which passed our criteria for significance but were not among the ones in LD with the original index SNPs in the reporting populations; SNP rs10965250 was reported in European population [47], and rs1111875 was reported in both European and Japanese populations [38], [79]–[81]. While these results may be novel associations, i.e. new signals in a region already implicated in GWAS studies, further replication (as in stage 2 of this two-stage GWAS study) will be needed before these will be well-accepted risk alleles.

It appears that our efforts to impute ungenotyped SNPs implemented by the programs SHAPEIT [70] and IMPUTE2 [72] were largely successful; as shown in the Results section we were able to impute with a high degree of estimated certainty for the large majority of ungenotyped risk alleles. We do note that the fraction of replicated risk alleles among imputed SNPs (6 of 35, 17.1%, Table S1) were smaller compared to the directly genotyped ones (9 of 19, 47.4%). This is partly explained by allele frequency and odds ratio differences which lead to somewhat decreased power (60.6% versus vs. 66.7%, Table S1) for imputed and genotyped SNPs respectively. In addition imputation involves some loss of power, governed by the *r^2^* between the imputed and true genotypes [82]. Nevertheless the score involving only imputed SNPs was a highly significant predictor of diabetes risk (p = 3.19×10^−8^).

More generally our findings indicate that only a very small fraction of T2D in Singapore Chinese can be explained by the SNPs in the risk regions examined to date. The rapid increase in T2D in Singapore and in other Asian and South East Asian communities [4], [21], [22] strongly indicate environmental factors are at play, yet susceptibility to these factors (notably BMI) appears to differ greatly by racial/ethnic group [83]–[85]. Understanding the interplay between genes and lifestyle-related risk factors that could produce such notable racial/ethnic disparities would seem to be among the most important needs in diabetes epidemiology. A separate report on genetic interactions between individual risk SNPs, genetic scores, and lifestyle or other “environmental” variables is under development using these data. It is clear also that very large sample sizes are needed to establish new T2D risk alleles since it is evident that each one plays a small role by itself even when strongly significantly predictive in composite (as in our risk score analyses). Our ability to extend through imputation the set of SNPs used in the present study (based on the Affymetrix Axiom ASI array) to over 7 million SNPs with good reliability and demonstrated predictive ability means that this study can contribute to the very large scale highly collaborative studies that may be needed to make further progress in understanding the genetics of T2D. Alternatively, significant differences may exist between ethnic groups, such that, the effect size of specific SNPs may differ between the ethnic groups as a result of differences due to early development and/or environment. In addition, the identification of less common SNPs (<5%) may be important and studies of T2D in ethnic groups would benefit from sequencing studies.

## Supporting Information

Figure S1 **Observed −log** ***P*** **compared to the corresponding power for each of the 54 reported T2D SNPs.** The reference solid line indicates observed *P* = 0.05.
(TIF)

Table S1 **Association results of Singapore GWAS for known T2D loci.**
(DOCX)
