# Supplementary figures and images for: Joint Effects of Known Type 2 Diabetes Susceptibility Loci in Genome-Wide Association Study of Singapore Chinese: The Singapore Chinese Health Study
Source: PLoS One. 2014 Feb 10;9(2):e87762. doi: 10.1371/journal.pone.0087762 (PMC3919750; doi:10.1371/journal.pone.0087762)

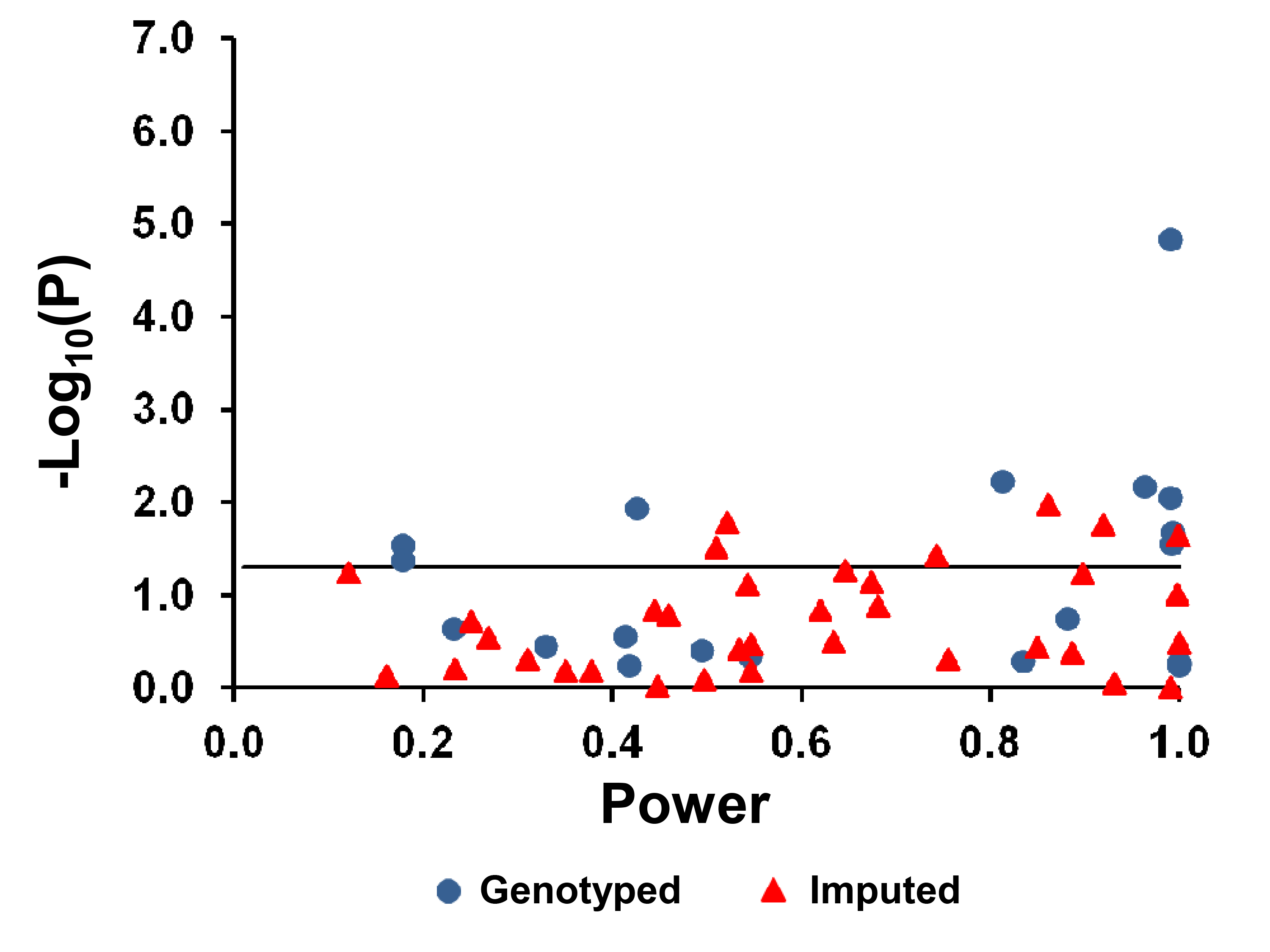

Supplement: Figure S1 — Observed −log P compared to the corresponding power for each of the 54 reported T2D SNPs. The reference solid line indicates observed P = 0.05. (TIF) [file pone.0087762.s001.tif]
