# Supplementary material for: Joint Effects of Known Type 2 Diabetes Susceptibility Loci in Genome-Wide Association Study of Singapore Chinese: The Singapore Chinese Health Study
Source: PLoS One. 2014 Feb 10;9(2):e87762. doi: 10.1371/journal.pone.0087762 (PMC3919750; doi:10.1371/journal.pone.0087762)
Supplement: Table S1 — Association results of Singapore GWAS for known T2D loci. (DOCX) [file pone.0087762.s002.docx]

**Table S1. Association results of Singapore GWAS for known T2D loci^1^**

| **SNPs** | **Data Type** | **CHR** | **Nearest Gene** | **Risk allele** | **Other allele** | **RAF**† **(Singa-pore)** | **GWAS Catalog Reporte Effect** | | |  | **Singapore GWAS** | | | | |
| --- | --- | --- | --- | --- | --- | --- | --- | --- | --- | --- | --- | --- | --- | --- | --- |
|  |  |  |  |  |  |  | **OR(CI)** | **P-value** | **Reference** |  | **OR(CI)** | **P-value** | **Power** | **Imp. r^2^‡** | **H-W P** |
| rs10965250 | Genotyped | 9 | CDKN2A/B | G | A | 0.586 | 1.2 (1.13-1.27) | 1.00E-10 | Voight BF, 2010 |  | 1.20 (1.12-1.28) | 1.48E-05 | 0.99 | NA | 7.63E-01 |
| rs10811661* | Genotyped | 9 | CDKN2A/B | T | C | 0.587 | 1.2 (1.14-1.25) | 8.00E-15 | Scott LJ, 2007 |  | 1.20 (1.12-1.28) | 1.84E-05 | 0.99 | NA | 1.00E+00 |
| rs2383208* | Genotyped | 9 | CDKN2A/B | A | G | 0.606 | 1.34 (1.27-1.41) | 2.00E-29 | Takeuchi F, 2009 |  | 1.15 (1.07-1.23) | 1.05E-03 | 1.00 | NA | 7.36E-01 |
| rs10906115 | Genotyped | 10 | CDC123 - CAMK1D | A | G | 0.618 | 1.13 (1.08-1.18) | 1.00E-08 | Shu XO, 2010 |  | 1.13 (1.04-1.21) | 5.88E-03 | 0.81 | NA | 1.83E-01 |
| rs9939609 | Genotyped | 16 | FTO | A | T | 0.129 | 1.25 (1.19-1.30) | 1.00E-20 | Perry JR, 2012 |  | 1.19 (1.01-1.36) | 6.80E-03 | 0.96 | NA | 1.92E-01 |
| rs7957197 | Imputed | 12 | OASL | T | A | 0.986 | 1.07 (1.05-1.10) | 2.00E-08 | Voight BF, 2010 |  | 1.96 (1.46-2.46) | 8.23E-03 | 0.08 | 0.51 | NA |
| rs1111875 | Genotyped | 10 | HHEX - EXOC6 | C | T | 0.298 | 1.21 (1.15-1.28) | 7.00E-12 | Takeuchi F, 2009 |  | 1.12 (1.04-1.21) | 8.76E-03 | 0.99 | NA | 7.46E-02 |
| rs5219 | Imputed | 11 | KCNJ11 | T | C | 0.351 | 1.14 (1.10-1.19) | 7.00E-11 | Scott LJ, 2007 |  | 1.12 (1.03-1.20) | 1.05E-02 | 0.86 | 0.99 | NA |
| rs5215* | Imputed | 11 | KCNJ11 | C | T | 0.351 | 1.14 (1.10-1.19) | 5.00E-11 | Zeggini E, 2007 |  | 1.12 (1.03-1.20) | 1.13E-02 | 0.86 | 0.99 | NA |
| rs7578326 | Genotyped | 2 | KIAA1486 - IRS1 | A | G | 0.853 | 1.11 (1.08-1.13) | 5.00E-20 | Voight BF, 2010 |  | 1.16 (1.04-1.27) | 1.16E-02 | 0.43 | NA | 1.93E-02 |
| rs831571 | Imputed | 3 | PSMD6 - PRICKLE2 | C | T | 0.623 | 1.09 (1.06-1.12) | 8.00E-11 | Cho YS, 2011 |  | 1.11 (1.01-1.21) | 1.63E-02 | 0.52 | 0.99 | NA |
| rs3802177 | Imputed | 8 | SLC30A8 | G | A | 0.537 | 1.15 (1.10-1.21) | 1.00E-08 | Voight BF, 2010 |  | 1.11 (1.02-1.19) | 1.74E-02 | 0.92 | 0.97 | NA |
| rs13266634* | Imputed | 8 | SLC30A8 | C | T | 0.539 | 1.22 (1.16-1.28) | 2.00E-14 | Takeuchi F, 2009 |  | 1.10 (1.02-1.19) | 1.96E-02 | 1.00 | 0.96 | NA |
| rs7756992 | Genotyped | 6 | CDKAL1 | G | A | 0.467 | 1.2 (1.13-1.27) | 8.00E-09 | Steinthorsdottir V, 2007 |  | 1.10 (1.00-1.20) | 2.08E-02 | 0.99 | NA | 6.38E-01 |
| rs3786897 | Imputed | 19 | PEPD | A | G | 0.565 | 1.1 (1.07-1.14) | 1.00E-08 | Cho YS, 2011 |  | 1.12 (1.02-1.22) | 2.19E-02 | 0.90 | 0.70 | NA |
| rs10440833 | Imputed | 6 | CDKAL1 | A | T | 0.354 | 1.25 (1.20-1.31) | 2.00E-22 | Voight BF, 2010 |  | 1.10 (1.00-1.21) | 2.25E-02 | 1.00 | 1.02 | NA |
| rs6931514* | Imputed | 6 | CDKAL1 | G | A | 0.470 | 1.25 (1.17-1.33) | 1.00E-11 | Zeggini E, 2008 |  | 1.10 (1.00-1.19) | 2.63E-02 | 1.00 | 1.02 | NA |
| rs6769511 | Genotyped | 3 | IGF2BP2 | C | T | 0.237 | 1.23 (1.15-1.31) | 1.00E-09 | Unoki H, 2008 |  | 1.11 (0.99-1.23) | 2.81E-02 | 0.99 | NA | 1.35E-01 |
| rs7766070* | Imputed | 6 | CDKAL1 | A | C | 0.352 | 1.21 (1.14-1.28) | 6.00E-11 | Perry JR, 2012 |  | 1.10 (1.00-1.20) | 2.83E-02 | 0.99 | 1.02 | NA |
| rs11642841 | Genotyped | 16 | FTO | A | C | 0.033 | 1.13 (1.08-1.18) | 3.00E-08 | Voight BF, 2010 |  | 1.29 (0.91-1.68) | 2.90E-02 | 0.18 | NA | 4.89E-01 |
| rs7178572 | Imputed | 15 | HMG20A | G | A | 0.343 | 1.09 (1.06-1.12) | 7.00E-11 | Kooner JS, 2011 |  | 1.10 (1.00-1.20) | 3.07E-02 | 0.51 | 0.99 | NA |
| rs1470579* | Genotyped | 3 | IGF2BP2 | C | A | 0.237 | 1.14 (1.09-1.19) | 2.00E-09 | Voight BF, 2010 |  | 1.11 (0.99-1.23) | 3.17E-02 | 0.78 | NA | 1.15E-01 |
| rs9470794 | Imputed | 6 | ZFAND3 | C | T | 0.340 | 1.12 (1.08-1.16) | 2.00E-10 | Cho YS, 2011 |  | 1.10 (0.99-1.20) | 3.71E-02 | 0.74 | 0.99 | NA |
| rs10923931 | Genotyped | 1 | NOTCH2 | T | G | 0.033 | 1.13 (1.08-1.17) | 4.00E-08 | Zeggini E, 2008 |  | 1.27 (0.90-1.63) | 4.22E-02 | 0.18 | NA | 1.62E-01 |
| rs6815464 | Imputed | 4 | MAEA | C | G | 0.659 | 1.13 (1.10-1.16) | 2.00E-20 | Cho YS, 2011 |  | 1.12 (1.01-1.23) | 4.48E-02 | 0.97 | 0.60 | NA |
| rs7172432 | Imputed | 15 | C2CD4A - C2CD4B | A | G | 0.681 | 1.11 (1.08-1.14) | 9.00E-14 | Yamauchi T, 2010 |  | 1.09 (1.00-1.18) | 5.38E-02 | 0.65 | 0.97 | NA |
| rs11634397 | Imputed | 15 | ZFAND6 - FAH | G | A | 0.081 | 1.06 (1.04-1.08) | 2.00E-09 | Voight BF, 2010 |  | 1.16 (0.95-1.38) | 5.72E-02 | 0.12 | 0.91 | NA |
| rs1333051 | Imputed | 9 | CDKN2A/B | A | T | 0.863 | 1.22 (1.15-1.30) | 6.00E-10 | Parra EJ, 2011 |  | 1.13 (1.00-1.25) | 5.83E-02 | 0.90 | 0.92 | NA |
| rs849134 | Imputed | 7 | JAZF1 | A | G | 0.776 | 1.13 (1.09-1.18) | 3.00E-09 | Voight BF, 2010 |  | 1.09 (1.00-1.19) | 7.24E-02 | 0.67 | 0.98 | NA |
| rs864745* | Genotyped | 7 | JAZF1 | T | C | 0.776 | 1.1 (1.07-1.13) | 5.00E-14 | Zeggini E, 2008 |  | 1.09 (1.00-1.19) | 7.29E-02 | 0.47 | NA | 1.42E-01 |
| rs6017317 | Imputed | 20 | FITM2 - R3HDML | G | T | 0.426 | 1.09 (1.07-1.12) | 1.00E-11 | Cho YS, 2011 |  | 1.08 (0.98-1.17) | 7.69E-02 | 0.54 | 0.99 | NA |
| rs4712524 | Imputed | 6 | CDKAL1 | G | A | 0.372 | 1.22 (1.15-1.31) | 3.00E-10 | Unoki H, 2008 |  | 1.07 (0.98-1.17) | 9.84E-02 | 1.00 | 1.02 | NA |
| rs7754840* | Genotyped | 6 | CDKAL1 | C | G | 0.372 | 1.12 (1.08-1.16) | 4.00E-11 | Saxena R, 2007 |  | 1.07 (0.98-1.17) | 9.87E-02 | 0.76 | NA | 7.97E-02 |
| rs10946398* | Genotyped | 6 | CDKAL1 | C | A | 0.372 | 1.16 (1.10-1.22) | 1.00E-08 | Zeggini E, 2007 |  | 1.07 (0.98-1.17) | 1.03E-01 | 0.94 | NA | 9.12E-02 |
| rs4402960* | Genotyped | 3 | IGF2BP2 | T | G | 0.230 | 1.14 (1.11-1.18) | 9.00E-16 | Scott LJ, 2007 |  | 1.08 (0.97-1.20) | 1.20E-01 | 0.77 | NA | 2.58E-02 |
| rs4689388 | Imputed | 4 | WFS1 | A | G | 0.901 | 1.16 (1.10-1.21) | 1.00E-08 | Rung J, 2009 |  | 1.19 (0.87-1.51) | 1.23E-01 | 0.84 | 0.37 | NA |
| rs7903146 | Imputed | 10 | TCF7L2 | T | C | 0.024 | 1.4 (1.34-1.46) | 2.00E-51 | Voight BF, 2010 |  | 1.23 (0.83-1.63) | 1.31E-01 | 0.68 | 0.99 | NA |
| rs7593730 | Imputed | 2 | RBMS1 | C | T | 0.838 | 1.11 (1.08-1.16) | 4.00E-08 | Qi L, 2010 |  | 1.09 (0.96-1.22) | 1.44E-01 | 0.45 | 0.98 | NA |
| rs1801214* | Imputed | 4 | WFS1 | T | C | 0.900 | 1.13 (1.08-1.18) | 3.00E-08 | Voight BF, 2010 |  | 1.18 (0.87-1.49) | 1.44E-01 | 0.69 | 0.37 | NA |
| rs7901695 | Imputed | 10 | TCF7L2 | C | T | 0.025 | 1.37 (1.31-1.43) | 1.00E-48 | Zeggini E, 2007 |  | 1.22 (0.82-1.61) | 1.46E-01 | 0.62 | 0.99 | NA |
| rs4506565* | Genotyped | 10 | TCF7L2 | T | A | 0.024 | 1.36 (1.20-1.54) | 5.00E-12 | WTCCC, 2007 |  | 1.22 (0.82-1.61) | 1.48E-01 | 0.60 | NA | 7.53E-01 |
| rs1535500 | Imputed | 6 | KCNK16;KCNK17 | T | G | 0.475 | 1.08 (1.05-1.11) | 2.00E-08 | Cho YS, 2011 |  | 1.06 (0.97-1.16) | 1.61E-01 | 0.46 | 0.86 | NA |
| rs231362 | Imputed | 11 | KCNQ1 | G | A | 0.868 | 1.08 (1.06-1.10) | 3.00E-13 | Voight BF, 2010 |  | 1.11 (0.92-1.31) | 1.71E-01 | 0.42 | 0.61 | NA |
| rs5015480 | Genotyped | 10 | HHEX - EXOC6 | C | T | 0.184 | 1.18 (1.13-1.23) | 1.00E-15 | Voight BF, 2010 |  | 0.93 (0.83-1.04) | 1.81E-01 | 0.88 | NA | 5.69E-02 |
| rs4712523* | Genotyped | 6 | CDKAL1 | G | A | 0.382 | 1.27 (1.21-1.33) | 7.00E-20 | Takeuchi F, 2009 |  | 1.06 (0.96-1.15) | 1.93E-01 | 1.00 | NA | 6.76E-02 |
| rs3923113 | Imputed | 2 | GRB14 - COBLL1 | A | C | 0.893 | 1.09 (1.06-1.13) | 1.00E-08 | Kooner JS, 2011 |  | 1.10 (0.96-1.24) | 1.93E-01 | 0.25 | 0.88 | NA |
| rs1802295 | Genotyped | 10 | VPS26A | T | C | 0.123 | 1.08 (1.05-1.12) | 4.00E-08 | Kooner JS, 2011 |  | 1.08 (0.93-1.22) | 2.28E-01 | 0.23 | NA | 5.43E-01 |
| rs7961581 | Genotyped | 12 | TSPAN8 - LGR5 | C | T | 0.222 | 1.09 (1.06-1.12) | 1.00E-09 | Zeggini E, 2008 |  | 1.06 (0.96-1.15) | 2.78E-01 | 0.41 | NA | 7.67E-01 |
| rs13292136 | Imputed | 9 | KRT18P24 - CHCHD9 | C | T | 0.918 | 1.11 (1.07-1.15) | 3.00E-08 | Voight BF, 2010 |  | 0.92 (0.77-1.07) | 2.83E-01 | 0.27 | 1.00 | NA |
| rs7041847 | Imputed | 9 | GLIS3 | A | G | 0.484 | 1.1 (1.07-1.13) | 2.00E-14 | Cho YS, 2011 |  | 1.04 (0.96-1.12) | 3.18E-01 | 0.63 | 0.98 | NA |
| rs391300 | Imputed | 17 | SRR | C | T | 0.657 | 1.28 (1.18-1.39) | 3.00E-09 | Tsai FJ, 2010 |  | 0.96 (0.88-1.04) | 3.25E-01 | 1.00 | 0.95 | NA |
| rs1387153 | Imputed | 11 | RPS3AP42 - MTNR1B | T | C | 0.462 | 1.09 (1.06-1.11) | 8.00E-15 | Voight BF, 2010 |  | 0.96 (0.88-1.04) | 3.30E-01 | 0.55 | 0.96 | NA |
| rs2237897 | Imputed | 11 | KCNQ1 | C | T | 0.682 | 1.33 (1.24-1.41) | 1.00E-16 | Unoki H, 2008 |  | 1.08 (0.93-1.23) | 3.38E-01 | 1.00 | 0.33 | NA |
| rs972283 | Genotyped | 7 | KLF14 - FLJ43663 | G | A | 0.679 | 1.07 (1.05-1.10) | 2.00E-10 | Voight BF, 2010 |  | 1.04 (0.95-1.14) | 3.51E-01 | 0.33 | NA | 6.39E-01 |
| rs2237892 | Imputed | 11 | KCNQ1 | C | T | 0.691 | 1.4 (1.34-1.47) | 2.00E-42 | Yasuda K, 2008 |  | 1.08 (0.92-1.23) | 3.52E-01 | 1.00 | 0.33 | NA |
| rs1359790 | Imputed | 13 | NDFIP2 - SPRY2 | G | A | 0.722 | 1.15 (1.10-1.20) | 6.00E-09 | Shu XO, 2010 |  | 1.05 (0.95-1.14) | 3.63E-01 | 0.85 | 0.91 | NA |
| rs12779790 | Imputed | 10 | CDC123 - CAMK1D | G | A | 0.197 | 1.11 (1.07-1.14) | 1.00E-10 | Zeggini E, 2008 |  | 1.05 (0.93-1.17) | 3.85E-01 | 0.53 | 0.84 | NA |
| rs4607103 | Genotyped | 3 | ADAMTS9 - MAGI1 | C | T | 0.667 | 1.09 (1.06-1.12) | 1.00E-08 | Zeggini E, 2008 |  | 1.04 (0.95-1.12) | 3.97E-01 | 0.50 | NA | 7.17E-01 |
| rs7305618 | Imputed | 12 | RPL12P33 - NCRNA00262 | C | T | 0.467 | 1.14 (1.09-1.20) | 2.00E-08 | Parra EJ, 2011 |  | 1.04 (0.95-1.12) | 4.12E-01 | 0.89 | 0.94 | NA |
| rs163182 | Imputed | 11 | KCNQ1 | C | G | 0.407 | 1.28 (NA) | 2.00E-17 | Cui B, 2011 |  | 0.95 (0.81-1.08) | 4.61E-01 | 1.00 | 0.32 | NA |
| rs4812829 | Genotyped | 20 | HNF4A | A | G | 0.446 | 1.09 (1.06-1.12) | 3.00E-10 | Kooner JS, 2011 |  | 1.03 (0.94-1.12) | 4.64E-01 | 0.55 | NA | 7.12E-02 |
| rs8090011 | Imputed | 18 | LAMA1 | G | C | 0.712 | 1.13 (1.09-1.18) | 8.00E-09 | Perry JR, 2012 |  | 1.03 (0.94-1.13) | 4.81E-01 | 0.76 | 0.98 | NA |
| rs1531343 | Imputed | 12 | RPSAP52 | C | G | 0.108 | 1.1 (1.07-1.14) | 4.00E-09 | Voight BF, 2010 |  | 0.95 (0.83-1.07) | 4.88E-01 | 0.31 | 0.99 | NA |
| rs515071 | Genotyped | 8 | ANK1 | G | A | 0.833 | 1.18 (1.12-1.25) | 1.00E-08 | Imamura M,2012 |  | 1.04 (0.92-1.15) | 5.13E-01 | 0.84 | NA | 2.27E-01 |
| rs17584499 | Genotyped | 9 | PTPRD | T | C | 0.107 | 1.57 (1.36-1.82) | 9.00E-10 | Tsai FJ, 2010 |  | 1.04 (0.90-1.19) | 5.30E-01 | 1.00 | NA | 8.19E-01 |
| rs243021 | Genotyped | 2 | EIF3FP3 - BCL11A | A | G | 0.663 | 1.08 (1.06-1.10) | 3.00E-15 | Voight BF, 2010 |  | 1.03 (0.93-1.12) | 5.68E-01 | 0.42 | NA | 1.17E-01 |
| rs1048886 | Genotyped | 6 | C6orf57 | G | A | 0.082 | 1.54 (1.32-1.80) | 3.00E-08 | Sim X, 2011 |  | 1.04 (0.88-1.21) | 5.73E-01 | 1.00 | NA | 3.79E-01 |
| rs896854 | Imputed | 8 | TP53INP1 | T | C | 0.252 | 1.06 (1.04-1.09) | 1.00E-09 | Voight BF, 2010 |  | 0.98 (0.88-1.07) | 6.20E-01 | 0.23 | 0.98 | NA |
| rs2943641 | Imputed | 2 | KIAA1486 - IRS1 | C | T | 0.930 | 1.19 (1.13-1.25) | 9.00E-12 | Rung J, 2009 |  | 1.04 (0.87-1.21) | 6.50E-01 | 0.55 | 1.00 | NA |
| rs1552224 | Imputed | 11 | ARAP1 | A | C | 0.930 | 1.14 (1.11-1.17) | 1.00E-22 | Voight BF, 2010 |  | 1.04 (0.88-1.20) | 6.53E-01 | 0.35 | 0.96 | NA |
| rs16861329 | Imputed | 3 | ST6GAL1 | C | T | 0.799 | 1.09 (1.06-1.12) | 3.00E-08 | Kooner JS, 2011 |  | 1.02 (0.92-1.12) | 6.63E-01 | 0.38 | 1.00 | NA |
| rs2237895 | Imputed | 11 | KCNQ1 | C | A | 0.359 | 1.29 (1.19-1.40) | 1.00E-09 | Tsai FJ, 2010 |  | 0.97 (0.83-1.11) | 6.82E-01 | 1.00 | 0.32 | NA |
| rs4457053 | Imputed | 5 | SNORA47 - PDE8B | G | A | 0.066 | 1.08 (1.06-1.11) | 3.00E-12 | Voight BF, 2010 |  | 0.97 (0.81-1.14) | 7.29E-01 | 0.16 | 0.99 | NA |
| rs6467136 | Imputed | 7 | ZNF800 - GCC1 | G | A | 0.812 | 1.11 (1.07-1.14) | 5.00E-11 | Cho YS, 2011 |  | 1.01 (0.91-1.12) | 8.10E-01 | 0.50 | 1.00 | NA |
| rs6780569 | Imputed | 3 | -- | G | A | 0.824 | 1.21 (1.14-1.30) | 6.76E-09 | Imamura M, 2012 |  | 1.01 (0.90-1.12) | 8.88E-01 | 0.93 | 0.99 | NA |
| rs2028299 | Imputed | 15 | AP3S2 | C | A | 0.192 | 1.1 (1.07-1.13) | 2.00E-11 | Kooner JS, 2011 |  | 1.00 (0.90-1.11) | 9.65E-01 | 0.45 | 1.01 | NA |
| rs7560163 | Imputed | 2 | RND3 - FABP5L10 | C | G | 0.881 | 1.33 (1.19-1.49) | 7.00E-09 | Palmer ND, 2012 |  | 1.00 (0.87-1.13) | 9.81E-01 | 0.99 | 0.91 | NA |
| rs7578597 | Genotyped | 2 | THADA | T | C | 0.992 | 1.15 (1.10-1.20) | 1.00E-09 | Zeggini E, 2008 |  | NA | NA | 0.13 | NA | 2.41E-01 |
| rs8042680 | Imputed | 15 | PRC1 | A | C | 0.998 | 1.07 (1.05-1.09) | 2.00E-10 | Voight BF, 2010 |  | NA | NA | 0.05 | 0.65 | NA |
| rs8050136* | Imputed | 16 | FTO | NR | C/A | 0.127 | 1.3 (1.23-1.39) | 2.00E-17 | Timpson NJ, 2008 |  | 1.20 (1.02-1.38) | 3.73E-03 |  | 0.98 | NA |
| rs7018475 | Genotyped | 9 | CDKN2A/B | NR | T/G | 0.355 | 1.35 (1.18-1.56) | 3.00E-08 | Huang J, 2012 |  | 1.13 (1.02-1.23) | 5.97E-03 |  | NA | 3.89E-01 |
| rs7656416 | Imputed | 4 | C4orf42 - MAEA | NR | C/T | 0.274 | 1.15 (1.10-1.21) | 1.00E-08 | Imamura M, 2012 |  | 0.89 (0.80-0.98) | 4.04E-02 |  | 0.67 | NA |

*SNPs were excluded from risk score because they are in close LD with one other SNP in the list.

†RAF represents the risk allele frequencies in our sample.

‡ r^2^ represents the imputation quality.

1. SNPs in red had inconsistent OR compare our results to published GWAS results.
